# Magnetic Nanoparticles Applications for Amyloidosis Study and Detection: A Review

**DOI:** 10.3390/nano8090740

**Published:** 2018-09-18

**Authors:** Jonathan Pansieri, Matthieu Gerstenmayer, François Lux, Sebastien Mériaux, Olivier Tillement, Vincent Forge, Benoit Larrat, Christel Marquette

**Affiliations:** 1 Laboratoire de Chimie et Biologie des Métaux, Université Grenoble Alpes, CNRS, CEA, 17 Rue des Martyrs, CEDEX 9, 38041 Grenoble, France; jonathan.pansieri@umu.se (J.P.); vincent.forge@cea.fr (V.F.); 2 NeuroSpin, CEA, Université Paris-Saclay, 91191 Gif-sur-Yvette CEDEX, France; matthieu.gerstenmayer@cea.fr (M.G.); sebastien.meriaux@cea.fr (S.M.); benoit.larrat@cea.fr (B.L.); 3 Université Lyon, Université Claude Bernard Lyon 1, CNRS, Institut Lumiére Matiére, F-69622 Lyon, France; francois.lux@univ-lyon1.fr (F.L.); olivier.tillement@univ-lyon1.fr (O.T.)

**Keywords:** magnetic nanoparticles, amyloidosis, Alzheimer’s diseases, targeted nanoparticles, medical imaging

## Abstract

Magnetic nanoparticles (MNPs) have great potential in biomedical and clinical applications because of their many unique properties. This contribution provides an overview of the MNPs mainly used in the field of amyloid diseases. The first part discusses their use in understanding the amyloid mechanisms of fibrillation, with emphasis on their ability to control aggregation of amyloidogenic proteins. The second part deals with the functionalization by various moieties of numerous MNPs’ surfaces (molecules, peptides, antibody fragments, or whole antibodies of MNPs) for the detection and the quantification of amyloid aggregates. The last part of this review focuses on the use of MNPs for magnetic-resonance-based amyloid imaging in biomedical fields, with particular attention to the application of gadolinium-based paramagnetic nanoparticles (AGuIX), which have been recently developed. Biocompatible AGuIX nanoparticles show favorable characteristics for in vivo use, such as nanometric and straightforward functionalization. Their properties have enabled their application in MRI. Here, we report that AGuIX nanoparticles grafted with the Pittsburgh compound B can actively target amyloid aggregates in the brain, beyond the blood–brain barrier, and remain the first step in observing amyloid plaques in a mouse model of Alzheimer’s disease.

## 1. Introduction

Amyloidoses can be defined as a large group of diseases characterized by soluble peptides or proteins aggregation in insoluble and very stable structures. They are composed of filamentous and unbranched architectures with β-sheets, so-called amyloid fibers. They are supplemented by small protein derivatives, made of two to six peptides or proteins, known as oligomers [1]. So far, in human pathology, 36 proteins have been identified to be amyloidogenic [2]. They are involved in a large panel of systemic, sporadic, or hereditary disorders with no structural or functional link. Obviously, their detection and characterization remain an important clinical challenge in various disorders for diagnosis and treatment. Various amyloid proteins are involved in metabolic diseases, such as β_2_-microglobulin [3] in hereditary visceral amyloidosis, calcitonin [4] in carcinoma of the thyroid, and islet amyloid polypeptide (IAPP) [5] in type 2 diabetes. Some other amyloid proteins can spontaneously form aggregates, such as fragments of apolipoproteins (ApoAI, ApoAII, ApoCII), with deposition in many organs and that are linked to atherosclerosis [6,7]. A third group of diseases related to amyloid deposits concerns neurodegenerative diseases, such as amyloid β (Aβ) in Alzheimer’s disease (AD), α-synuclein (α-syn) in Parkinson’s disease (PD), or prion protein (PrP) in Creutzfeldt–Jakob disease, which are the most studied amyloidoses due to their major clinical impacts [8]. Alzheimer’s disease affects 47 million people around the world [9] and is characterized by a progressive cognitive impairment without reliable early diagnosis nor efficient treatment. Moreover, one of the main issues concerning these neurodegenerative diseases comes from the limited ability of drugs to reach the brain due to the blood–brain barrier (BBB), which is the physiological barrier that separates blood vessels from cerebral parenchyma [10].

In this context, in the last twenty years, nanotechnologies has become a very promising field in modern medicine in general and in the amyloidosis field in particular. Indeed, a large diversity of organic and inorganic functionalized nanomaterials is already used in clinical research [11]. The development of nanoparticles (NPs) at the nanometer scale (typically 10–300 nm) has become a front-line tool because of their high versatility in terms of composition, shape, size, and functionalization. This opens up new possibilities in a number of applications, such as disease therapy and disease diagnosis, including magnetic resonance imaging. The combination of magnetic properties and controlled surface functionalization allows for the design of sensing nanoparticles for analytes [12,13] (DNA and protein target detection), pathogens [14], such as bacteria and circulating tumoral cells [15], and amyloid peptides or protein aggregates [16]. Recently, magnetic NPs (MNPs) have also become interesting tools by integration into transducer materials using an external magnetic field for active detection on various surfaces. Thus, MNPs and a wide range of properties of molecules may be combined to increase the sensitivity of sensors as magnetic field devices, piezoelectric devices [17], electrochemical biosensors [18,19], and optical devices [20,21]. Concerning amyloidosis, NPs are promising biosensors as imaging agents for early diagnosis or therapeutic follow up [22] or as drug carriers with specific amyloid inhibitors with controlled release [23].

Magnetic nanoparticles (MNPs) were notably highlighted [24] because of their intrinsic magnetic properties and remain very useful tools for non-invasive MRI. In addition, some MNPs have shown potential properties to overcome certain biological and physical barriers, such as the BBB [25], and have proved their usefulness with tissue repair and hyperthermia properties [26]. MNPs gather superparamagnetic iron oxide nanoparticles (SPIONS), NPs with a metallic core (cobalt, zinc, nickel …), and other nanoparticles coated with magnetic material (silica NPs coated by gadolinium chelates or with iron …) [27]. In the framework of in vivo use, MNPs need to be properly designed to conciliate in situ detection by medical imaging, biological barrier crossing, and other transport restriction in biological tissues. Thus, fundamental requirements need to be fulfilled [28]: a biocompatibility with no or minimal toxicity for patients with a minimal accumulation within the liver; total body elimination of MNPs through the kidney; and the ability to reach their target through blood circulation while keeping their intrinsic magnetic and targeting properties. For example, the MNPs’ size is an important factor for their ability to cross cells and biological barriers and is more often inversely related to their potential in vivo toxicity [29]. Even if this parameter depends on the MNP’s metallic atom, charge, and functionalization, it is believed that <10 nm particles are the best for intravenous (i.v.) administration [29,30].

**Figure 1:**
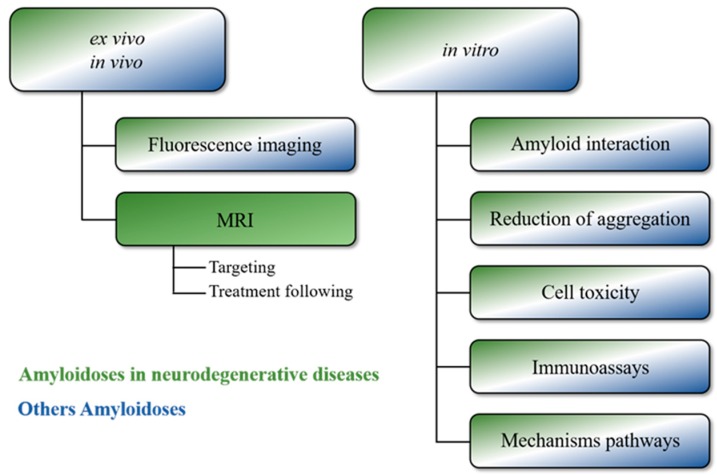
Principal insights of magnetic nanoparticles (MNPs) applications in other amyloidoses fields.

In this review, we summarize MNPs designed against various amyloidosis-related diseases for biomedical applications, taking into account that neurodegenerative diseases are more often priority targets. This purpose includes MNPs as detection platforms by sensitive imaging techniques mostly implicated in the fundamental understanding of amyloid aggregation as well as in amyloid detection and quantification for diagnosis and as an indicator for therapy follow up (Figure 1).

## 2. MNPs for the In Vitro Investigation of Amyloid Deposition Mechanisms

**Figure 2:**
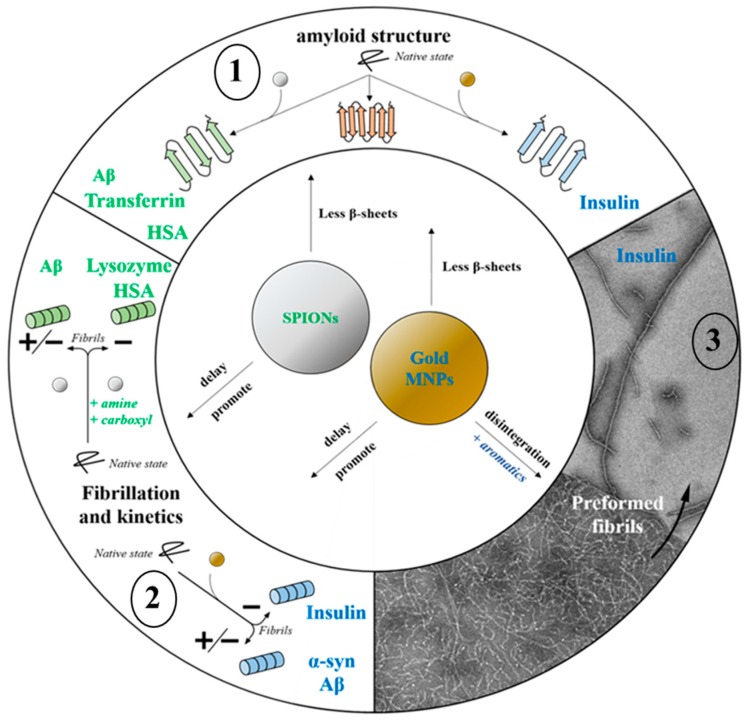
MNPs impacts on various in vitro amyloid aggregates. According their size, shape, charge, and type of functionalization, MNPs can: (**1**) affect amyloid structure at the protein level by decreasing β-sheet content; (**2**) inhibit, delay, or promote protein fibrillation and elongation into fibers; and (**3**) promote disassembly of amyloid fibers. HSA, human serum albumin.

The formation mechanisms of amyloid aggregates are highly complex and still widely studied. Nowadays, the simplified phenomenon of nucleation-polymerization proposed in the 1980s [31] seems to be supplemented by several concomitant processes proposed in the literature [32,33,34,35,36,37]. Even if all amyloid fibers share a common β-sheet structure, the pathways leading to their formation are numerous. Fibrillogenesis and oligomerization can be initiated by denatured proteins that are fully or partially folded, preformed fibers (seeding), and/or by amorphous aggregates. Moreover, the mechanisms and kinetics of aggregation are closely linked. They depend on various favorable factors (temperature, concentration, pH …) and on the diversity of interaction mechanisms (co-aggregation of different amyloid proteins, seeding effect, metal ion presence …) [36]. Thus, a better understanding of these mechanisms can be helpful for the diagnosis and the treatment of these disorders and can be related to disease incidences. Interestingly, MNPs without amyloid-specific functionalization constitute efficient tools to study, in vitro, amyloid protein aggregation by controlling the self-assembly of amyloid proteins. Indeed, MNPs may delay, inhibit, or increase amyloid deposition and there is great potential to understand their mechanisms of action as precursors in therapeutic applications (Figure 2).

### 2.1. MNPs for the Understanding of Aβ Fibrillation

Iron oxide NPs, especially Ultrasmall SuperParamagnetic Iron Oxides (USPIOs) and SPIONs of 10–150 nm diameter [38], show an impact on various amyloid proteins’ aggregation, especially Aβ. These MNPs are made of a core shell of magnetite (Fe_3_O_4_), maghemite (γ-Fe_2_O_3_), or a combination of both, generally coated with biocompatible polyethylene glycol (PEG). This PEG coating acts as a shield against the hydrophobic properties of some cells while helping NP synthesis, but Cheng et al. showed that it might be indirectly responsible for increased cytotoxic effects due to linkage with 1-éthyl-3-(3-diméthylaminopropyl)-carbodiimideN-hydroxysuccinimide (EDC-NHS) [39]. However, MNPs may disturb the formation mechanisms, kinetics, and cytotoxicity of amyloid aggregates by directly affecting their structures [40,41,42,43,44,45,46,47]. Thus, highly concentrated, positively charged PEGylated-SPIONs accelerate Aβ aggregation, whereas ones of low concentration delay it [40,41]. Otherwise, at the same high concentration, negatively charged USPIOs promote less Aβ fibrillation. These interesting results could be useful to investigate Aβ mechanism pathways (control of Aβ fibrils’ nucleation and elongation thanks to the dual effect of SPIONs coated with positive charges) and for clinical use (a small number of side effects from negatively charged SPIONs) [40]. A previous study confirms that low-concentration MNPs may be a critical factor for in vivo applications by enhancing Aβ fiber formation with titanium oxide NPs (TiO_2_NPs) [47]. Moreover, MNP conjugates can play a protective function for cells, as has been shown by using SPIONs–heparin, which reduces the toxicity of Aβ aggregates in vitro for neuronal cells with a paradoxical acceleration of Aβ fiber formation [43]. These results suggest that MNPs can change the Aβ amyloid aggregates’ conformation to a less toxic pathway without avoiding their aggregation.

In a different way, MNPs have a great potential to investigate oligomer formation mechanisms and to measure in vivo oligomer levels [48], the oligomers being the most toxic form of amyloid deposits [49]. The hydrophobic environment of gold nanoparticles (AuNPs) can be used as an interface to indirectly design specific oligomer antibodies [50] by mimicking Aβ oligomerization of the spherical amyloid oligomers on their surface. A combination of an Aβ peptide with TiO_2_NPs is able to enhance Aβ oligomers production thanks to an equilibrium between TiO_2_NPs–Aβ interaction and Aβ oligomers found in solution [47]. In this case, MNPs could be useful to investigate the mechanisms of amyloid oligomers production.

### 2.2. MNPs for the Control of Various Amyloidogenic Proteins’ Assembly

The most frequent outcome of studies on various amyloid proteins follows the same ascertainments, with an effect on nucleation and growth rate with or without an effect on preformed fibers. Hsieh et al. shows that AuNPs can affect insulin fibril formation by delaying fibrillation and modifying the elongation of fibers to make them much shorter and more compact [51]. These results suggest that MNPs can disturb the self-assembly process by acting directly on the β-sheet core. In addition, their functionalization with aromatic aminoacids may destabilize these insulin fiber architectures and may even trigger their disassembly in solution [52]. In contrast, similar kinetics assays, performed with AuNPs on α-syn proteins, showed faster and higher fibrillation inversely dependent on the nanoparticle size [53].

SPIONs also disturb hen egg white lysozyme fibrillogenesis, slowing nucleation and amyloid fibril elongation, and reduce the toxicity of neuroblastoma and fibroblast cells [42]. Using Thioflavin T (ThT) fluorescence measurements, another study showed that MnFe_2_O_4_ MNPs functionalized with amine and carboxylate drastically inhibit human serum albumin (HSA) fibrillation [54]. Indeed, the reduction of ThT fluorescence intensity indicates a decrease in β-sheet content in HSA fibers, as this molecule is known to interact with cross-β-sheets and thus is used for experimental amyloid aggregation detection. Skaat et al. go further and show that fluorinated γ-Fe_2_O_3_ MNPs inhibit insulin aggregation by delaying the α-helix to β-sheet structure transition due to their strong hydrophobic character [55]. Thus, MNPs can change also the morphology of amyloid fibers by acting directly on monomers or the protein structure as shown by irreversible changes in transferrin conformation, an amyloid protein involved in several disorders [45,46]: addition of SPIONs affects directly the protein folding that occurs before reaching the pathological forms.

## 3. MNPs Functionalization for the Detection of Amyloid Aggregates

Nowadays, MNPs are used as biosensors for fundamental research and preclinical applications. The basic idea is to use their magnetic properties for their detection, strengthened by coupling with amyloid ligands on MNP surfaces, to target an amyloid burden. A large panel of ligands already exists and various detection techniques can be applied (spectroscopy, electrochemiluminescence [56], ELISA, fluorescence imaging …) thanks to MNPs’ versatility.

### 3.1. Congo Red and Thioflavin Derivatives Dyes

Congo Red and the previously mentioned polar molecule ThT were the first known molecules tested for the in vitro detection of amyloid deposits. They are able to inhibit a lot of the formation of oligomers and fibers while stabilizing the monomeric and partially folded forms. In this context, iron oxide NPs doped with Thioflavin have been recently synthesized, but have not yet been fully tested on amyloid aggregates [57]. Indeed, these molecules have barely been used for grafting onto MNPs for medical purposes as Congo red is potentially toxic in vivo and some chemical modifications permit us to increase the affinity and stability of ThT. However, 2-(4′-[^11^C]methylaminophenyl)-6-hydroxybenzothiazole, one of ThT’s neutral derivatives, the Pittsburgh compound B (PIB), constitutes one of the best targeting molecules for the detection of various amyloid deposits [58]. Thus, to date, the ^11^C-PIB probe is the standard agent for AD diagnosis by PET imaging [59]. However, studies have reported that unspecific ^11^C-PIB labelling is detected in the brain of healthy patients, raising the question of its efficiency for early diagnosis [60]. Recently, Gd^3+^-based silica NPs functionalized with PIB (called Gd(DOTA)PEG-PIB) have been shown to interact with various amyloid fibers, i.e., the Aβ, IAPP, and transthyretin (TTR) amyloid fibers, and spectroscopy measurements of PIB fluorescence display affinities that remain 3 to 4 orders of magnitude [16] below those of free PIB or ^11^C-PIB used in PET imaging [61].

### 3.2. Peptides Derived from Amyloid Proteins

In order to add a specific recognition of an amyloid protein, numerous research projects synthesized MNPs grafted with amyloid proteins or a short peptide derived from protein sequences. Monocrystalline iron oxide NPs (MIONs) and Gd^3+^-based NPs grafted with the entire Aβ_1–40_ protein [62] show great affinities for Aβ deposits by ELISA, with dissociation constant (KD) values in the 100-nM range. However, this type of grafting obviously accelerates the fibrillation [55]. Thus, shorter peptides from amyloid sequences make them more suitable for economical and straightforward grafting and have a small immunogenic reaction [63,64]. This type of peptide can be selected by phage display to target amyloid aggregates, and most studies involve Aβ amyloid proteins. A large number of them directly comes from the β-sheet structure buried in their hydrophobic core. AuNP conjugates with CLPFFD-NH2 adhere to Aβ fibers with good affinity and stability [65], as do fluorinated LPFFD–iron oxide NPs conjugates, supplemented in this case by an inhibition of Aβ fibril formation, with a delay of up to three days [55,66]. Moreover, we showed a specific recognition of Aβ fibrils and deposits by Gd-based MNPs conjugates (Gd(DOTA)PEG) grafted with the peptide KLVFF or the peptide LPFFD using fluorescence spectroscopy and fluorescence imaging on AD mice tissues [67]. Their specificity toward Aβ deposits was confirmed by demonstrating their lack of interaction with other types of amyloid fibers, i.e., TTR and IAPP fibers.

Interestingly, Xia et al. go further by developing a network of silver nanoparticles (AgNPs) grafted with PrP(95–110) peptides, and used them as redox reporters to specifically detect the oligomer form of Aβ by high-sensitivity electrochemistry [68]. The method consists of using a AgNPs network and a cellular prion protein (PrPc), a specific receptor of Aβ oligomers. PrPc is coupled with adamantine (Ad) on a β-cyclodextrin electrode’s surface, which produces an electrochemical signal that is drastically amplified thanks to the redox reaction Ag/AgCl due to AgNPs. When Aβ oligomers interact with the Ad–PrPc surface, the electrochemical signal significantly decreases because less AgNPs are binding. This interesting work, with a limit of detection below 10 pM, could be used to precisely quantify Aβ oligomers in cerebrospinal fluid in the case of AD [69].

### 3.3. Antibodies and Nanobodies

Composites with anti-mouse IgG on AuNP surfaces may be used as a sensitive in vitro immunosensor platform, providing a great potential tool for clinical early Parkinson’s disease (PD) detection thanks to the analysis of low abundant α-syn deposits [70]. Another study goes further, using SPIONs grafted with monoclonal antibodies against α-syn, and permits us to differentiate PD dementia from PD by the evaluation of plasma α-syn [71]. In the same way, Wang et al. designed an immunosensor based on zinc MNPs, functionalized with both luminol and an anti-Aβ antibody, to specifically detect Aβ aggregates by electrochemiluminescence [56]. Others show that MIONs coated with the antibody anti-Aβ_42_ can specifically detect and bind to cerebrovascular Aβ deposits in leptomeningeal vessels in an AD mice model, following their injection, and they could be detected in vivo by high-field-strength magnetic resonance imaging [72].

However, the size of antibodies is important and grafting them on small MNPs remains a critical issue for crossing the BBB and in view of envisaging their translation to the brain and clinical applications. Therefore, nanobodies, the monomeric variable domain of the antibody (12–15 kDa), have been set up to address these issues. Nowadays, numerous nanobodies selected by phage display and directed toward various amyloid deposits are available: B10AP against Aβ, Nb23 against β_2_-microglobulin, and cAb-HuL6 against lysozyme [73,74,75,76,77]. Indeed, nanobodies provide a lot of benefits: they are easy to produce, they are constituted by one single variable domain (VHH), they are less immunogenic and have a smaller size than antibodies while keeping their high affinity for amyloid deposits, and have a better chance to cross biological membranes [68,77]. Consequently, we have grafted the specific nanobody (B10AP) [78] to Gd(DOTA)PEG, and we showed that this complex displayed a high affinity for Aβ fibers in vitro, with KD values in the 10-nM range and specific Aβ targeting on ex vivo AD mice model brain sections [16].

### 3.4. MNPs for Other Amyloidosis Detection Tests

Although much of this research field is focused on Alzheimer’s Disease, some studies obviously show benefits for other medical applications. Pal et al. describe the detection of the β_2_-microglobulin and ApoA1 amyloid proteins by polyclonal antibodies–MNPs in serum, with usefulness for the detection of ovarian cancer in an early stage [79]. The nanobody B10AP grafted with Gd(DOTA) shows detection towards amyloid fibers of islet amyloid polypeptide (IAPP) and fibers of transthyretin (TTR), which are involved in type 2 diabetes and polyneuropathy, respectively, and has great affinities with KD values in the 10-nM range in vitro [16]. Fluorescence imaging on slices of pancreas and stomach tissues of mice models using Gd(DOTA)-Cy5.5-B10AP has also shown selective targeting of IAPP and TTR aggregates [16].

## 4. MNPs as In Vivo Diagnostic Probes

The current technique for the detection and quantification of amyloidosis in vivo is positron emission tomography (PET) using radiolabeled probes presenting high and specific affinity for amyloid aggregates. In particular, brain amyloidosis can be staged with this technique and this new information can be combined with cognitive scores to confirm the diagnosis of AD and allow for accurate stratification of the disease for better patient management. Nowadays, several tracers, i.e., Amyvid (^18^F-Florbetapir), Vizamyl (^18^F-Flutemetamol), and Neuraceq (^18^F-Florbetaben), have obtained Food Drug Administration approval as amyloid PET imaging probes and are currently used in the clinic [80,81,82,83]. PET can quantify the amyloid burden, but its low spatial resolution does not allow for visualization of single amyloid plaques. Positive scans using these probes do not establish a diagnosis of Alzheimer’s disease, or other cognitive disorder; they increase the likelihood that memory impairment is caused by Alzheimer’s disease. Furthermore, the lack of in vivo binding validation of these probes and the consequent deficiency in the understanding of their tissue binding and specificity are genuine concerns; neither compound is approved to quantify amyloid plaques in the brain. As a consequence, these tracers are an adjunct to other diagnostic evaluations. To improve specificity, a large number of other tracers are under clinical development. The literature contains highly divergent reports of the binding selectivity of these agents for the different forms of Aβ; for example, [^11^C]PIB is often claimed to be able to bind strongly to fibrillar Aβ but only weakly to diffuse Aβ plaques. However, a number of recent studies have observed significant [^11^C]PIB retention in correlation with the presence of both fibrillar and diffuse Aβ plaques, leading authors to assert that this tracer cannot differentiate neuritic from diffuse amyloid plaques [84]. To conclude, none of the studies performed with these PET tracers mapped out Aβ pathologies in sufficient detail to allow for quantitative regional cortical correlation of an amyloid imaging signal with an underlying pathology throughout the whole brain irrespective of the neurodegenerative disease. Magnetic resonance imaging (MRI) is also in fast development for medical diagnosis of neurodegenerative diseases in general and AD in particular. This technique produces two- and three-dimensional high-resolution images that do not involve ionizing radiation, which is required by radiochemistry. In the clinical routine, AD diagnosis is mainly done by observation of cerebral atrophy (medial temporal structures), which occurs at relatively late stages of the disease when cognitive decline has already started. More recently, functional alterations have also been investigated as early indirect and largely unspecific biomarkers using techniques such as task-based or resting state blood oxygen level dependent functional MRI.

**Figure 3:**
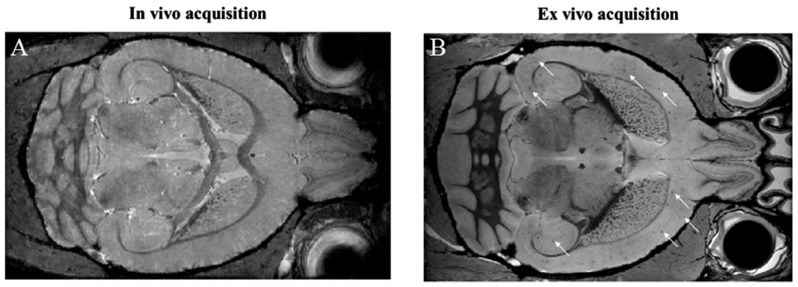
Alzheimer’s disease (AD) mouse brain (12 months old) T_2_*-weighted MRI images acquired at 7T without a contrast agent. No direct amyloid plaque detection is possible in vivo (**A**) while the absence of blood and the highest resolution make amyloid plaques clearly visible ex vivo (**B**). Three-dimensional (3D) gradient-echo sequences: In vivo parameters: echo time 4 ms, repetition time 90 ms, echo spacing 3 ms, resolution 60 × 60 × 60 µm^3^, matrix size 280 × 180 × 120, number of echoes 8, no average, acquisition time 32 min; Ex vivo parameters: echo time 4 ms, repetition time 90 ms, echo spacing 4.75 ms, resolution 40 × 40 × 40 µm^3^, matrix size 400 × 270 × 180, number of echoes 8, 12 averages, acquisition time of 12 h.

Efforts are engaged in an earlier diagnosis of the disease, and MRI is currently being investigated as a unique tool to achieve in vivo amyloid plaque imaging [85]. Indeed, a large proportion of amyloid plaques stores iron in their cores and therefore modifies the MRI signal in their surroundings, giving hypo-intense spots (a susceptibility effect) on T_2_ and T_2_* images [86,87,88]. The main problem that remains is that the required acquisition time for reaching a sufficiently high resolution (100 µm) to detect these spots is much too long (>2 h) for applying the technique to weak patients in a clinical routine even at a high magnetic field. However, this can be achieved in small animal models at a very high magnetic field with high performance sensors and long acquisition times allowing for very high resolution (<100 µm) [89]. Even under such conditions, this technique works well ex vivo after exsanguino-perfusion of the brains [90] while it remains difficult to perform in vivo as shown in Figure 3. This is because of the relatively lower spatial resolution achieved in vivo in reasonable scan times and also because of the competition between T_2_ hypo-signals originating from blood iron and iron contained in plaques.

Thus, the development of contrast agents is crucial for distinguishing AD tissue and improving amyloid imaging. Some authors have tested the possibility of using untargeted gadolinium-based MNPs to enhance the contrast between the amyloid plaques and the surrounding brain tissue, a technique known as “passive staining” [91]. It consists in delivering Gd-based contrast agents (Gd-DOTA, Dotarem**^®^**) to the whole brain by intra-cerebro-ventricular injections in vivo or by a bath in a contrast agent solution for fixed brains. However, the clinical translation of this approach seems difficult for several reasons: in vivo homogeneous delivery of Gd chelates into the brain by ventricular injection is not possible in patients and the contrast-to-noise ratio remains low under in vivo imaging conditions, making amyloid plaques difficult to detect from blood vessels as the blood also contains iron.

In order to increase the specificity and contrast-to-noise ratio of amyloid detection, targeted MNPs have been developed and validated for their capability to bind amyloid aggregates. The availability of MRI amyloid tracers can provide an earlier diagnosis of neurodegenerative diseases but also a quantitative monitoring of treatments. Two main types of MNPs are currently used to detect amyloid deposits: superparamagnetic contrast agents and paramagnetic contrast agents. The first category, i.e., iron oxide NPs, such as USPIO, MION, and SPION, acts as T_2_ contrast agents that decrease locally the image signal by shortening T_2_, while the second category, i.e., Gd-derived NPs, acts as T_1_ contrast agents that enhance locally the image signal by shortening T_1_. In the case of iron oxide NPs, MNPs have been grafted with Aβ peptide moieties, such as Aβ_1–40_ or Aβ_1–42_, K6Aβ_1–30_, or antibody fragments, such as anti-Aβ_42_-F(ab’)2. Peptides target amyloid plaques, and MNP properties show a significant decrease in apparent transverse relaxation (T_2_*) values [62,92,93]. However, in these studies a toxicity was reported, which was likely due to Aβ peptides grafted on MNPs or to the immunogenicity of targeting antibodies, which can constitute a limit for their clinical translation. This important aspect needs to be taken into account from the beginning of the development of these targeted MNPs. A large variety of SPIONs has been designed for MRI because they present a certain efficiency to cross the BBB and act as promising in vivo contrast agents [92,93,94,95,96,97,98,99]. On AD mice models, Anti-AβPP antibodies-coated SPIONS doubled the number of MRI-visible plaques with suggested spontaneous crossing of the BBB [94]. Poduslo et al. shows specific dark spots on T_2_*-weighted images located in cortical arterioles thanks to monoclonal antibody IgG4.1-targeted MIONS [72]. Some other iron oxide NPs grafted with peptides having a hydrophobic character and selected through a library of small peptides, termed USPIO-PHO, remain a high contrast tool for transgenic mice using T_2_-weighted MRI sequences [86,87,88,89,90,91,92,93,94,95,96,97,98]. MNPs grafted with the polyphenolic curcumin, and stabilized by an amphiphilic copolymer, have shown great detection of amyloid deposits in ex vivo brain scanned by T_2_*-weighted MRI [39]. Similar work with the anti-Aβ monoclonal antibody BAM10 grafted to γ-Fe_2_O_3_ nanoparticles shows that they are able to interact with Aβ_40_ fibrils and to target Aβ plaques ex vivo in rat brain detected by MRI imaging [99]. These SPION-based MNPs present encouraging results in animal models but their utilization in humans remains limited owing to many drawbacks [100]. Mainly, the dark spots due to the blood vessels are often indistinguishable from the ones due to SPION-targeted amyloid aggregates. Therefore, proper amyloid diagnosis still needs further ex vivo confirmation [101]. As an example, using USPIO-PEG-Aβ_1–42_ [92], authors reported hypo-signals similar to a compound targeting amyloid in wild-type (WT) mice, emphasizing the difficulty in distinguishing marked amyloid plaques from blood vessels on T_2_* images. To avoid confusion with blood vessels, a contrast agent affecting T_1_ relaxation might be useful, as it would induce hyper intense signals that are easily distinguishable from blood vessels.

Manganese oxide NPs grafted with anti-Aβ_1–40_ antibodies show hyper-enhanced spots in the frontal cortex area with T_1_-weighted images [95]. Manganese ions shorten the T_1_ relaxation times of surrounding protons and were proposed as in vivo paramagnetic probes that are easily distinguishable from blood hypo-intense signals. However, this family of MNPs is restricted to animal studies since manganese ions are toxic in the brain.

**Figure 4:**
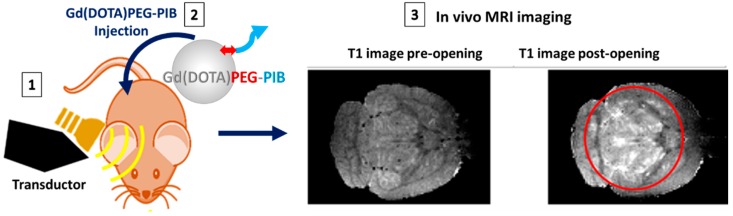
Ultrasound technology was applied via a transductor (**1**) on the brain of a transgenic mouse model of Alzheimer’s disease (APP/PS1 ΔE9) in order to increase the delivery of Gd(DOTA) grafted with PIB (Gd(DOTA)PEG-PIB) previously intravenously injected (**2**). The method consists of sonicating a large part of the brain, allowing for BBB disruption with a global intensity and on a high volume of brain, as observed in the post-ultrasound image compared to the prefrontal cortex (~+20%): MRI images show T_1_-weighted images pre- and post-BBB opening along with injection of Gd(DOTA)PEG-PIB (**3**). The MNPs signal appears as a hypersignal affecting the T_1_ relaxation time.

Alternatively, Gadolinium chelates are the most widely used MRI contrast agents, with nine products already approved for clinical applications [102]. Gd^3+^ ions also reduce the T_1_ relaxation time of surrounding protons. Some, i.e., AGuIX**^®^**, show high r_1_ relaxivity due to their high paramagnetism [103], stability, and clearance from the body [104]. Regarding AD and cerebral amyloid angiopathy, the combination of Gd-based NPs with several targeting molecules, i.e., PIB, polyphenols, peptides, and antibodies, remains the common strategy [98,104,105,106,107,108]. Most of these works attempt to prove the in vivo concept, but the concept also needs ex vivo confirmation. Thus, in AD mice models, a putrescine-gadolinium-Aβ probe (PUT-Gd-Aβ [109]) is able to improve the contrast of amyloid plaques on T_1-_weighted images with a weak, but significant, contrast between plaques and the tissue in vivo. Gd(DO3A)-PIB shows a specific recognition of Aβ plaques [61], as do Gd(DTPA) grafted with Aβ peptide [67] or curcumin [106] and anti-Aβ antibody IgG4.1 vectorized on Gd(DOTA) [107]. Likewise, AGuIX NPs were grafted with PIB probes (Gd(DOTA)PEG-PIB) for the diagnosis of amyloidoses. These MNPs are made of a polysiloxane network surrounded by several DOTAGA(Gd^3+^) chelates [108,109]. AGuIX nanoparticles have a hydrodynamic diameter smaller than 5 nm and have been already efficiently used for MRI imaging and radio-sensitization [110]. Free ligands are available at the surface of AGuIX and can be used to chelate radioisotopes (e.g., ^68^Ga^3+^ or ^111^In^3+^) in order to perform PET or Single-photon emission computed tomography (SPECT) imaging [111,112]. Moreover, the covalent grafting of a near-infrared dye, such as cyanine5.5 (Cy5.5), is also achievable for optical imaging [113]. Recently, we showed that AGuIX can be functionalized with specific Aβ peptides to selectively target and image Aβ-amyloid fibrils [67]. Similarly, these nanoparticles grafted with PIB, Gd(DOTA)PEG-PIB, showed effective and specific interaction with amyloid plaques in vitro. Therefore, they were injected in vivo for evaluating MRI amyloid imaging and targeting (Figure 4). As those compounds present moderate efficiency to cross naturally the BBB, we have mechanically increased its permeability using an ultrasound-induced BBB opening (see Supplementary Materials). In this context, a low-intensity focused ultrasound are a safe and reproducible technique to transiently open the BBB for drug delivery [114,115,116]. Microbubbles, intravenously injected in the blood, will oscillate under the action of the ultrasound and mechanically stress the endothelial cells of the BBB, which will contract and loosen their tight-junctions. This opening lasts few hours and allows for particles up to 60 nm (hydrodynamic diameter) to cross the BBB [117]. We have used this ultrasound technology to target amyloid plaques in vivo with PIB-grafted Gd(DOTA)PEG, which are small MNPs (<5 nm), to increase their delivery ((1) in Figure 4). The MRI images in Figure 4(3) show T_1_-weighted images pre- and post-BBB opening along with injection of Gd(DOTA)PEG-PIB. Ultrasound were shot, by a piezoelectric transducer, in the brain of a double transgenic mouse model of Alzheimer’s disease (APP/PS1 ΔE9) [118]. We used a global sonication of the brain to cover a large volume as evidenced by the signal enhancement in the sonicated area on the post-ultrasound image compared to the non-sonicated prefrontal cortex (~+20%). These results suggest that the ultrasound protocol was efficient in opening the BBB and allowed the MNPs to reach the brain parenchyma.

**Figure 5:**
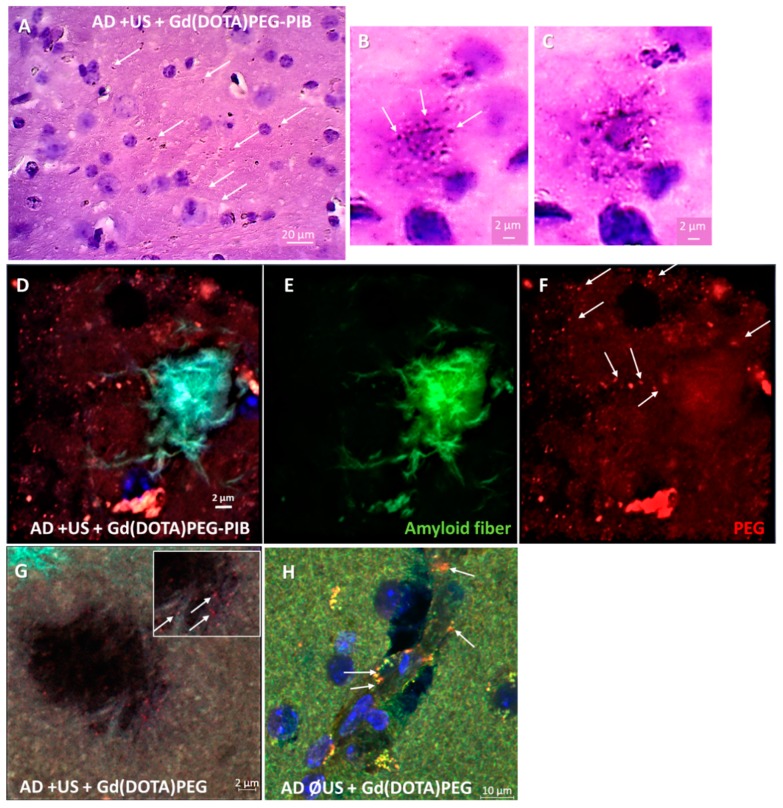
Immunohistological pictures of Gd(DOTA)PEG-PIB delivery inside cerebral tissue after blood–brain barrier (BBB) opening by ultrasound. (**A**–**C**) The images revealed the diffusion of MNPs into the brain using an antibody directed against the spacer PEG integrated in MNPs (brown spots) (**A**). Some of the Gd(DOTA)PEG-PIB were located in the vicinity of amyloid plaques (hematoxylin–eosin counterstaining) (**B**,**C**). (**D**–**F**) Confocal microscopy observations confirmed a close colocalization (**D**) between amyloid fibers ((**E**): thioflavin S staining (green)) and Gd(DOTA)PEG-PIB ((**F**): anti-PEG antibody revealed by secondary conjugated antibody Alexa-594 (red)). (**G**,**H**) For control, other AD mice were injected with MNPs without the amyloid-targeting PIB with (**G**) or without (**H**) prior ultrasound opening of the BBB; MNPs diffuse into the brain, but no specific accumulation around amyloid aggregates was observed (**G**), suggesting they remained localized in the vessel (**H**).

Thereafter, the ability of nanoparticles to target amyloid plaques in vivo was assessed by immunohistology (Figure 5; see method in Supplementary Materials). Gd(DOTA)PEG-PIB has been found in brain tissue, mainly in the thalamus nuclei (ventral, lateral, and posterior lateral), geniculate nuclei, amygdaloid (basolateral, post-lateral, post-medium) nuclei, and piriform and enthorinal cortex (Figure 5A). Some nanoparticles appear to be associated with or close to the amyloid aggregates (Figure 5B–F), suggesting the need to follow the behavior of the MNPs at different times after injection to distinguish specific from nonspecific targeting. Control nanoparticles (without the PIB ligand) also cross the BBB after ultrasound and diffused into the tissue (Figure 5G). However, no colocalization was evidenced between amyloid plaques and Gd(DOTA)PEG. Finally, the nanoparticles injected into mice whose BBB was not opened by the ultrasound technique remained in the cerebral vascular area (Figure 5H).

## 5. Conclusions

Functionalized or free MNPs remain accurate and versatile tools to better understand amyloidosis, especially concerning neurodegenerative diseases. Nowadays, they have become essential objects in the search for new medical approaches to amyloid deposits in fundamental and applied research. The ability to detect them by MRI remains a major advantage thanks to their intrinsic magnetic properties. Efforts should be made to ensure the safety of their use [119] in applications ranging from proteomics and genomics screening [120] to pathophysiology assessments. In this context, a growing number of MNPs is used nowadays in the research field, and careful consideration should be given in terms of their toxicity for human health and for the environment. Another potential limitation of this MRI-based strategy for repeated chronic injections in patients is the potential toxic accumulation of metals in the brain. Indeed, the clearance mechanisms of iron and gadolinium from the brain are still poorly known and this topic requires extensive study. Therefore, thanks to advances in bioengineering, the production of biocompatible MNPs with high medical relevance appears to be one of the most promising ways to diagnose and treat various amyloidosis associated with a wide range of diseases.

## Supplementary Materials

Materials and Methods are available online at http://www.mdpi.com/2079-4991/8/9/740/s1.

## Abbreviations

MNPs	magnetic nanoparticles
PIB	Pittsburgh compound B
AD	Alzheimer’s Disease
PD	Parkinson’s Disease
Aβ	amyloid beta protein
α-syn	α-synuclein
PrP	prion protein
PrPc	cellular prion protein
IAPP	islet amyloid polypeptide
TTR	transthyretin
BBB	blood–brain barrier
MRI	magnetic resonance imaging
PET	positron emission tomography
PEG	polyethylene glycol
USPIO	ultrasmall paramagnetic iron oxide
MION	magnetic iron oxide nanoparticles
SPION	supramagnetic iron oxide nanoparticles
ThT	Thioflavin T
i.v.	intravenous administration
