# Supplementary material for: Magnetic Nanoparticles Applications for Amyloidosis Study and Detection: A Review
Source: Nanomaterials (Basel). 2018 Sep 18;8(9):740. doi: 10.3390/nano8090740 (PMC6164038; doi:10.3390/nano8090740)
Supplement: Supplementary file 1 [file nanomaterials-08-00740-s001.pdf]

Supplementary Informations

Mice preparation and MRI setup

The homemade ultrasound setup consisted in a single element spherically focused concave and MR compatible piezoelectric transducer (diameter of 25 mm, focal depth 20 mm, central frequency at 1.5 MHz, Imasonic, France), mounted on a 3 axis motor stage. The transducer was driven by a portable generator and amplifier (Image Guided Therapy, Pessac, France).The emitted acoustic field was characterized both in a water tank and with simulations. The transducer was coupled to the head of the animals with a latex balloon filled with deionized and degassed water. The mice used in this study were two years old male APP/PS1ΔE9 transgenic mice, a model that exhibit amyloid plaques from 6 months of age.

Two mice were anesthetized with isoflurane (2-3% in an air-oxygen mix, 1L/min) and their head were shaved to ensure a good coupling with the transducer via a water tank. They were placed in the cradle of the MRI bed under the ultrasound transducer. Then, 100μL of microbubbles (diameter 2-8 μm, Sonovue, Bracco) were retro-orbitary injected. Ultrasound were immediately shot continuously for 5 minutes. During sonication, the transducer was moved over the head of the mice so that the focal spot covers a large volume of the brain, as described by Magnin *et al.* [1]. The estimated peak negative pressure in the brain was 0.6 MPa. Immediately after the BBB disruption, the MNPs were intravenously injected and mice were placed inside the MRI (11.7 Tesla, Bruker, Germany) equipped with a cryo-probe. Temperature and breathing rate were monitored. T_1 weighted images (MSME, 2D, TE/TR = 6/290 ms, resolution 130x130x250 μm, matrix size 140x110x28, 5 averages, acquisition time 2.5 minutes) were acquired to detect to MNPs in the brain. T_1 weighted images (same parameters) were previously acquired before the ultrasound protocol to be used as a reference.

Detection of amyloid plaques and peptide by immunohistochemistry.

At the end of MRI acquisition and imaging time lasted for not more than 2 hours, mice were anesthetized for intracardiac perfusion of 10 ml of phosphate buffered saline followed by 10 ml of physiological saline solution containing 4% paraformaldehyde. At the end the brains were removed and harvested in 4% paraformaldehyde for 48 hours. Brains were then cut using a microtom and floating slices were recuperate on polylysine slides conserved at -20°C. The presence of targeting nanoparticles in the brains of transgenic mice were observed by immunohistological techniques. For this purpose, endogenous peroxydases were blocked 10 min with dual endogenous enzyme block (S2003, Dako) and rinsed three times for 5 min in PBS 1X. Brain slices were pre-incubated with blocking buffer (PBS 1X- Triton 0.3%-BSA 1%) for1 h before incubation with rabbit anti-polyethylene glycol antibody (1/500, #ab133471, Abcam) for 1h30. After rinsing three times for 5 min in PBS 1X, slices were incubated for 1h at room temperature with peroxidase-conjugated goat anti-rabbit antibody (1/1000, #G-21234, Thermofisher). Nanoparticles were then detected by treating tissues for 20 min with 0.05% 3,3'-diaminobenzidine tetrachlorhydrate (Dako). After rinsing again three times in distilled water, the sections were counterstained with hematoxylin solution (HHS16, Sigma) and eosin Y solution (HT110116, Sigma), dehydrated and mounted in a permanent medium Eukitt (ChemLab). Images were obtained using a Zeiss microscope Axiovert 200 M equipped with Axiocam ERc 5s camera (Carl Zeiss) and analyzed using Carl Zeiss AxioVision software.

Other frozen sections were processed for fluorescence immunohistochemistry with same anti PEG antibody and with Thioflavin S for amyloid aggregates as previously described [16]. Thereby, Gd(DOTA)-PEG-PIB nanoparticles targeting amyloid plaques was evaluated through detection the fluorescence of secondary antibody conjugated with Alexa 594 (λex = 590 nm - λem = 617 nm) and amyloid burden with ThS (λex = 450 nm - λem = 488 nm) (Sigma, #T1892) according to standard practice. Nuclei were revealed by Hoechst dye. Fluorescent images were recorded using Zeiss confocal LSM 800 microscope and analyzed using Carl Zeiss Zen software.

Reference

1. Magnin, R.; Rabusseau, F.; Salabartan, F.; Mériaux, S.; Aubry, J.-F.; Le Bihan, D.; Dumont, E.; Larrat, B. Magnetic resonance-guided motorized transcranial ultrasound system for blood-brain barrier permeabilization along arbitrary trajectories in rodents. *J. Ther. Ultrasound* **2015**, *3*, 22, doi:10.1186/s40349-015-0044-5.
